# Role of Hepatitis C Virus Envelope Glycoprotein E1 in Virus Entry and Assembly

**DOI:** 10.3389/fimmu.2018.01411

**Published:** 2018-06-19

**Authors:** Yimin Tong, Dimitri Lavillette, Qingchao Li, Jin Zhong

**Affiliations:** ^1^Unit of Viral Hepatitis, CAS Key Laboratory of Molecular Virology and Immunology, Institut Pasteur of Shanghai, Chinese Academy of Sciences, Shanghai, China; ^2^Unit of Interspecies Transmission of Arboviruses and Antivirals, CAS Key Laboratory of Molecular Virology and Immunology, Institut Pasteur of Shanghai, Chinese Academy of Sciences, Shanghai, China; ^3^University of Chinese Academy of Sciences, Beijing, China

**Keywords:** hepatitis C virus, envelope protein, E1, virus entry, virus assembly, fusion

## Abstract

Hepatitis C virus (HCV) glycoproteins E1 and E2 form a heterodimer to constitute viral envelope proteins, which play an essential role in virus entry. E1 does not directly interact with host receptors, and its functions in viral entry are exerted mostly through its interaction with E2 that directly binds the receptors. HCV enters the host cell *via* receptor-mediated endocytosis during which the fusion of viral and host endosomal membranes occurs to release viral genome to cytoplasm. A putative fusion peptide in E1 has been proposed to participate in membrane fusion, but its exact role and underlying molecular mechanisms remain to be deciphered. Recently solved crystal structures of the E2 ectodomains and N-terminal of E1 fail to reveal a classical fusion-like structure in HCV envelope glycoproteins. In addition, accumulating evidence suggests that E1 also plays an important role in virus assembly. In this mini-review, we summarize current knowledge on HCV E1 including its structure and biological functions in virus entry, fusion, and assembly, which may provide clues for developing HCV vaccines and more effective antivirals.

## Introduction

Hepatitis C virus (HCV) is a major human pathogen that currently infects about 170 million people worldwide. Although recent introduction of highly effective direct-acting antiviral agents has greatly improved hepatitis C treatment outcome, no prophylactic HCV vaccine is available, rendering it difficult to eradicate HCV infections globally (1, 2). HCV is an enveloped, positive-strand RNA virus belonging to the family of *Flaviviridae*. The HCV RNA genome is 9.6-kb in length and encodes a single polyprotein that is co- or post-translationally cleaved into three structural proteins (core, E1, and E2) and seven non-structural proteins (p7, NS2, NS3, NS4A, NS4B, NS5A, and NS5B) (1). The envelope glycoproteins E1 and E2 form a stable heterodimer that mediates virus entry and morphogenesis. HCV virions are associated with host low-density lipoproteins or very-low-density lipoproteins, which play important roles in virus entry, egress, and evasion of the host immune response (3). HCV entry and morphogenesis are highly coordinated processes, which involve all viral structural and non-structural proteins as well as a panel of host factors (4–6). Here, we aim to summarize current knowledge of HCV E1 including its structure and biological functions in virus entry and morphogenesis.

## Structure of E1

### Domains/Motifs Organization

**Figure 1:**
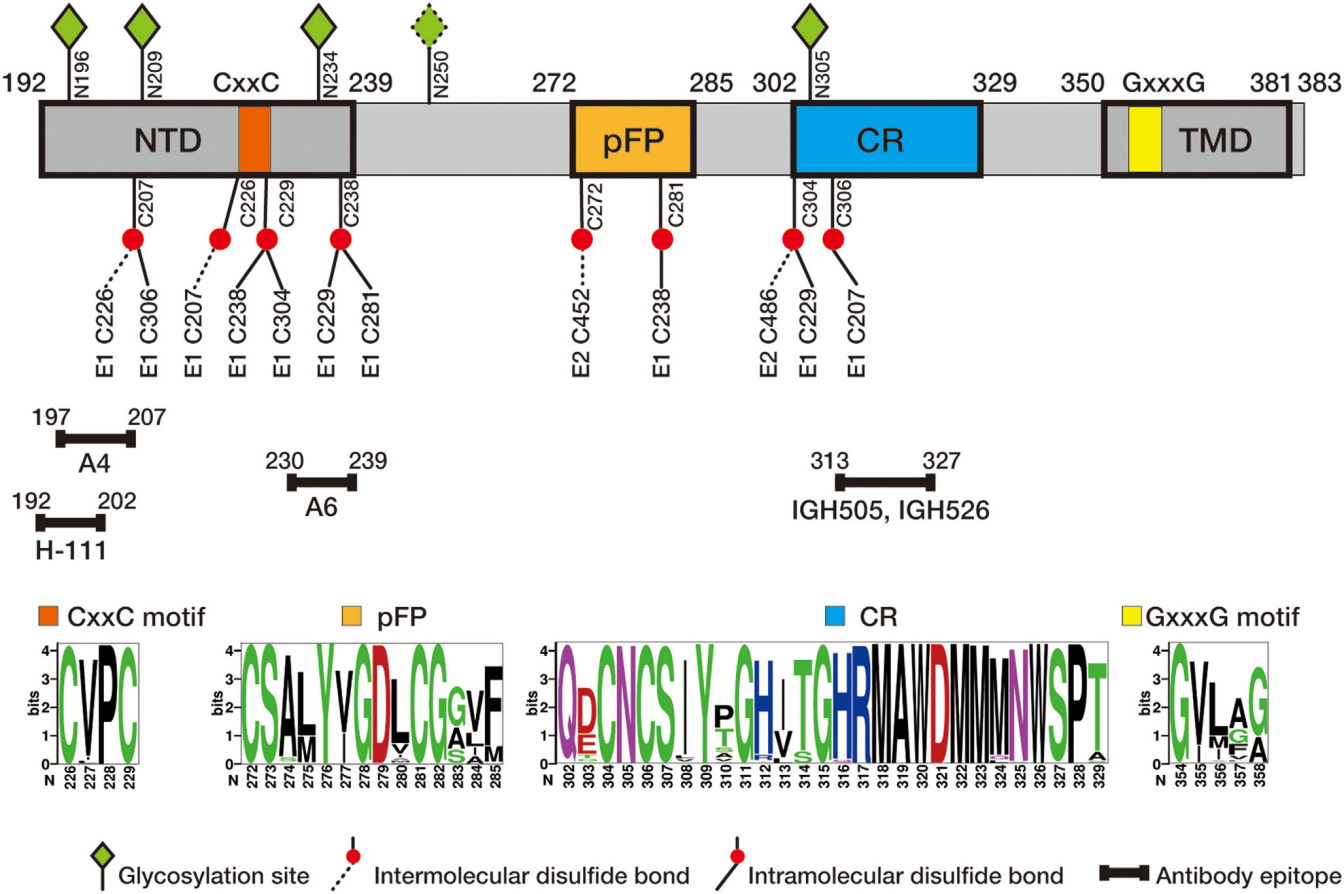
Schematic diagram of hepatitis C virus (HCV) E1 envelope protein. E1 contains N-terminal domain (NTD, 192–239), putative fusion peptide (pFP, 272–285), conserved region (CR, 302–329), and C-terminal transmembrane domain (TMD, 350–381). CxxC motif in NTD and GxxxG motif in TMD are marked in orange and yellow, respectively. Four N-glycosylation sites (N196, N209, N234, and N305) conserved in all genotypes and one genotype 1b/6-specific site (N250) are labeled in green diamond, and conserved cysteine residues are labeled in red ball (intra- and intermolecular disulfide bounds in solid and dash line, respectively). Antibody epitopes, recognized by antibodies H-111 (9), A4 (10), A6 (11), IGH505, and IGH526 (12, 13), are marked with line segments. Of them H-111, IGH505, and IGH526 have been shown to neutralizing viral infection. The numbers correspond to the position of amino acid residues in the HCV polyprotein, with the first residue of E1 starting at 192. Sequence logos were generated based on 184 E1 sequences of genotype 1–6 from the Los Alamos hepatitis C sequence database (14) using WebLogo (15).

E1 envelope glycoprotein (192 amino acids) is much smaller than E2 (approximately 365 amino acids depending on the genotypes) but both are type I transmembrane protein with the N-terminal ectodomain residing in the endoplasmic reticulum (ER) lumen and the C-terminus anchoring on the ER membrane. The length of HCV E1 and E2 is similar to that of pestivirus E1 and E2, but many other flaviviruses only encode a single envelope glycoprotein E of 500 amino acids. Bioinformatics analysis of E1 sequences across all genotypes reveals a conserved protein domain organization, including N-terminal domain (NTD, 192–239), putative fusion peptide (pFP, 272–285), conserved region (CR, 302–329), and C-terminal transmembrane domain (TMD, 350–381) (Figure 1). NTD contains four conserved cysteines that form intramolecular, and possibly intermolecular, disulfide bonds. In addition, majority of E1 glycosylation sites and identified E1 epitopes reside in this domain, suggesting NTD is likely exposed on the protein surface. The exact roles of NTD remain elusive, although it was shown that a motif (aa 219–221) in NTD may have a cross talk with TMD to determine the complex formation with E2 (7). TMD, also serving as the signal peptide for E2, dictates membrane-bound topology of E1 and is essential for forming a heterodimer with E2. pFP is highly conserved and has been proposed to participate in fusion of viral envelope and host cell membrane during HCV entry (5, 8). CR is highly conserved among all genotypes/subtypes, but its function is poorly defined.

### Glycosylation

Both E1 and E2 are heavily glycosylated, and N-linked oligosaccharides are added to asparagine (Asn) within the context sequon Asn-X-Ser/Thr (16). E1 of major genotypes possesses four conserved potential N-linked glycosylation sites (196, 209, 234, and 305) (17), while the fifth glycosylation site at N250 is only specific to genotypes 1b and 6 (18). E1 is not efficiently glycosylated if expressed alone, and requires the co-expression of E2 protein for the full-extent glycosylation (16).

E1 glycosylation contributes to correct protein folding and its biological functions. An early study showed that mutation of N196 or N305 impairs the E1/E2 heterodimerization while mutation of N209 or N234 has little effect (17). A later study based on HCV pseudoparticles (HCVpps) confirmed that glycosylation at N196 or N305 is critical for E1 folding and its incorporation into HCVpp, whereas N209 may modulate the virus entry (19). Using cell-culture derived HCV (HCVcc) system, it was shown that N196 is the most critical among the four E1 glycosylation sites for the HCVcc infectivity (20). In addition, E1 glycosylation pattern may regulate the formation of disulfide bond in E1. For example, disulfide bond involving C306 will likely be prevented by glycosylation at N305 due to spatial restriction (17). Interestingly, removal of this glycosylation increases the immunogenicity of soluble E1 (21). Another study also found that removal of the glycan at N209 improves immunogenicity of the E1/E2 heterodimer (22).

### Disulfide Bonds

Eight cysteine residues (C207, C226, C229, C238, C272, C281, C304, and C306) are highly conserved across all HCV genotypes. Although extensive analyses have been performed to decipher the possible disulfide bond matches among these cysteines, these efforts only yielded limited and conflicting information thus far. The solved partial E1 crystal structure revealed an intramolecular disulfide bond between C229 and C238 as well as an intermolecular disulfide bond between C207 and C226 (23). However, this finding is contrasted by another structure modeling study suggesting C226 remains in a free thiol state (24). Instead, this study proposed three different intramolecular disulfide bonds C207–C306, C229–C304, and C238–C281 (24). Moreover, possible disulfide bonds between E1 and E2 have also been proposed based on the proximity of cysteines in the predicted E1/E2 structures, such as C272 (E1) and C452 (E2), C304 (E1), and C486 (E2) (24, 25). These conflicting reports not only reflect the technical difficulty to determine the existence of disulfide bonds but also may reflect the complexity in dynamic changes of disulfide bond formation in E1 during the processes of HCV entry and morphogenesis. Indeed, virion-associated E1 and E2 envelope glycoproteins formed large covalent complexes stabilized by disulfide bridges, whereas the intracellular forms of these proteins assembled as noncovalent heterodimers (26). In addition, C226–C229 form a classical CxxC motif, a key feature of protein disulfide isomerase, which may mediate the isomerization of disulfide bonds in E1 during virus entry (24, 27), as reported in Env fusion proteins from retroviruses (28, 29). More experiments are needed to validate this hypothesis, as reports using thiol-reactive agents indicated that HCV entry is weakly dependent on its redox status, in contrast to other enveloped viruses (30). Mutagenesis of the eight conserved cysteines in E1 indicated that, unlike cysteine mutations in E2 that drastically disrupt virus infectivity (31), the cysteine mutations in E1 have much less effect on virus infectivity (27). Interestingly, the E1 cysteine mutant viruses hardly survive from the freeze-thaw treatment that normally does not harm wild-type HCVcc, suggesting that the disulfide bonds in E1 are more flexible but critical for maintaining stability of infectious virions (27).

### Crystal Structure

The NMR structures of partial E1 domains are available, including the region 314–342 (structure 2KNU) (32) that overlaps with the CR domain, and the region 350–369 (structure 1EMZ) (33) that resides in the TMD. The crystal structure of N-terminal domain of E1 (192–270, nE1) was recently solved (23). The nE1 monomer structure contains an N-terminal β-hairpin, a 16 amino acid α-helix in the middle and a three-strand antiparallel β-sheet in the C-terminal. Six nE1 monomers form an asymmetric unit, stabilized by a series of intra- and intermolecular disulfide bonds. Given that the nE1 crystals were prepared at a low pH condition, the covalently linked nE1 dimer may represent the post-attachment conformation of E1 formed in an acidic endosomal compartment. Interestingly, the six-stranded β-sheet structure formed by the nE1 homodimers appears similar to that of phosphatidylcholine transfer protein (23), raising a possibility that this domain may be one of the structural elements of HCV envelope proteins to mediate the lipoprotein association during HCV morphogenesis. Unfortunately, this solved nE1 structure gave little structural insight into membrane fusion as the majority of pFP was missing in the structure. The authenticity of this truncated E1 structure still needs to be validated experimentally, and future efforts should be focused on analysis of the full-length E1 ectodomain structure and E1 structure in complex with E2.

### E1 Oligomer and E1E2 Heterodimer

Oligomeric status of the global HCV envelope protein complex may fluctuate during the HCV replication cycle. Using HCVcc system, it was shown that trimeric E1 can be detected at the surface of virions by SDS-PAGE under reducing and mild thermal denaturation conditions (34). The formation of trimeric E1 requires the co-expression of E2, and the C-terminal TMDs on the both E1 and E2 appear sufficient to trigger the E1 trimerization. The highly conserved GxxxG motif (Gly354 and Gly358) located in the N-terminal of E1 TMD is critical for the formation of the E1 trimer (34). Interestingly, unlike the trimeric E1, E2 remains a monomer in SDS-PAGE under the same mild thermal denaturation condition. A working model proposes that the TMDs of three E1 monomers form a trimer in the center and simultaneously interact with the TMD of peripheral E2 to form a heterodimer (24, 34). It is unclear whether the conformation of trimeric E1E2 heterodimers is unique to the mature viral particles since this thermal-instable E1 trimer can be also detected in the lysate of infected cells (34).

Expression of E1 and E2 alone can lead to formation of a noncovalent heterodimer, which is retained in the ER inside the cell (35, 36). E1/E2 heterodimerization is critically dependent on interaction between their TMDs which consist of a single α-helix (33, 35, 37).

Truncation or mutation in this α-helix abolishes heterodimerization (38). The result of alanine scanning assay demonstrated that the TMDs consist of charged residues in their centers that act as ER retention signals and are directly involved in heterodimerization (39, 40). Mutagenesis studies show that the residues G354, G358, and Lys 370 in N-terminal of E1 TMD are essential for heterodimerization (33, 41).

## The Role of E1 in Attachment and Binding during Virus Entry

Hepatitis C virus envelope glycoproteins bind to specific proteins at the surface of hepatocytes to initiate the entry process. This process involves a surprisingly large number of host receptors/co-receptors/factors, and also confers the major determinant of viral tropism (4). These host receptors/co-receptors/factors have been well summarized by recent reviews (2, 4–6). Of them, scavenger receptor BI (SR-BI), cluster of differentiation 81 (CD81), and two tight junction proteins claudin-1 (CLDN1) and occludin1 (OCLN) play the most critical role in HCV entry, and thus are regarded as the real viral receptors/co-receptors. A recent single viral particle imaging analysis on the polarized cell culture revealed a sequential engagement of these receptors/co-receptors during HCV entry which involves the translocation of HCV from the initial contact site on the basolateral membrane to the tight junction (42).

E2 is the major HCV envelope protein that directly interacts with the receptors/co-receptors. The physical interactions between E2 and CD81, SR-BI have been biochemically demonstrated, sometime even in the absence of E1 (43, 44). It is long believed that the role of E1 in this process is mainly to assist E2 by maintaining a functional E2 conformation required for the receptor binding. Indeed, it was showed that the E1E2 complex can interact with CLDN1 whereas E2 alone cannot (45). Consistently, two independent studies showed that mutations in E1 can shift the usage of HCV entry factor from CLDN1 to CLDN6 (46, 47), highlighting the importance of E1 in interaction with CLDN1 during HCV entry process. Furthermore, a critical cross talk between E1 and E2 was identified to modulate E1E2 binding to HCV entry receptors SR-BI and CD81 (45). Interestingly, recent studies suggested that the role of E1 in virus attachment and binding appears more than just assisting E2. A study showed that E1, but not E2, binds ApoE and ApoB, the apolipoproteins that are decorated on HCV virions and are crucial for HCV entry through low-density lipoprotein receptor (48). However, this observation was contradicted by a later study showing that E2 instead of E1 interacts with ApoE (49). Another study showed that E1 directly binds CD36 to facilitate HCV attachment (50).

## The Role of E1 in Membrane Fusion

Endocytosis takes place upon the engagement of HCV envelope proteins with the receptors. It is well believed that the acidic environment in endosome activates the conformational changes of the envelope proteins and triggers the fusion of viral lipid envelope and endosomal membrane, leading to release of HCV RNA genome to cytoplasm (51). Despite extensive research, the molecular mechanism underlying the membrane fusion during HCV entry remains obscure. For a long time, the key unanswered questions were which viral protein(s) serve the fusion function and how the E1E2 heterodimer changes the conformation to induce membrane fusion.

The E glycoprotein of flaviviruses, a well-characterized prototype of class II fusion protein, consists of three distinct domains (DI, DII, and DIII), containing a fusion peptide buried at the dimer interface at neutral pH (8) and carrying both binding and membrane fusion properties (6). HCV E2 was initially considered as the viral fusion protein because of its size as well as its major role in receptor binding. However, the recent solved HCV E2 core structure exhibits a compact and Ig-like pattern (52, 53), which is different from any class II viral fusion protein-like structures shared by other flaviviruses (8), ruling out the possibility that E2 alone serves as fusion protein. This was further supported by the resolution of E2 structure of BVDV-1, a pestivirus member of the Flaviviridae family (8, 54).

It is now believed that E1 of BVDV and HCV serves as the fusion protein. E1 contains a conserved hydrophobic sequence (CSALYVGDLC, residues 272–281), which has been proposed to be a pFP (55). A number of studies demonstrate that this domain is indeed involved in the HCV fusion process (56–61). In addition, E1 can form a trimer, a typical structure of all fusion proteins. However, E1 seems too small to have a known class II or class III fold that could connect cellular and viral membranes after the fusion peptide insertion. Therefore, HCV E1 may define a new class of membrane fusogen. Interestingly, E1 of Rubella virus in the *Togaviridae* family does not possess the structural features of a classic class II fusion protein, while E1 of alphaviruses in the same *Togaviridae* family harbors a typical class II fusion protein (62). We speculate that constraints on flaviviruses or alphaviruses imposed by alternating life cycles between vertebrates and arthropods may result in more conservative evolution of their fusion proteins than for hepacivirus and rubivirus that infect human only. In the absence of this constraint, the structure of HCV or Rubella virus E1 may have evolved a number of specific features, placing it apart from classical class II fusion proteins of known structure.

Rather than being mediated by a single glycoprotein, HCV fusion appears to be mediated by complex intra- and intermolecular E1E2 dialogs that shape structural and conformational rearrangements of the heterodimer complex, similar to rubivirus and alphavirus (63). Consequently, the characterization of interplays between E1 and E2 is critical to decipher the HCV fusion (45). By combining computational analysis and wet-lab data, it was suggested that E1 co-evolves with the Back Layer domain (BL) of E2, and this genetic association is critical for membrane fusion (64). A soluble BL-derived polypeptide inhibits fusogenic rearrangements and HCV infection, suggesting E1 and E2 BL/Stem regions govern HCV fusion in a concerted manner (64).

## The Role of E1 in HCV Morphogenesis

Compared to virus entry, much less studies have been conducted to address how E1 contributes to HCV morphogenesis. It is believed that the formation of E1E2 heterodimer is a prerequisite for assembly of HCV virion. Any mutations that interfere with the dimerization of E1 and E2 would have a severe impact on HCV morphogenesis. For example, the mutations in the GxxxG motif located in TMD of E1 can disrupt the trimerization of E1 and formation of the E1E2 heterodimer, which further prevents the assembly of appropriate tertiary and quaternary structures (25, 34). In addition, E1 and E2 in virions are linked covalently by disulfide bridges (26), suggesting that HCV envelope proteins undergo conformational changes involving disulfide bond modification during virus assembly process.

E1 or the E1E2 complex can interact with NS2 (65, 66), the master viral protein that interacts with multiple viral structural and non-structural proteins to coordinate HCV assembly. This raises a possibility that E1 directly contributes to HCV morphogenesis in a way that may not involve E2. A mutation D263A in E1 abolishes the viral infectivity and leads to secretion of viral particles devoid of genomic RNA (47). Because the direct contact of E1 and viral genome is unlikely, it is tempting to speculate that E1 may regulate the assembly of infectious virions through its interaction with other viral proteins such as NS2.

We recently developed a trans-complementation-based HCV reverse genetics model in which the coding sequence for E1 or E1E2 is deleted from the HCV genome and is provided *in trans* (57, 67). This system allows to perform the mutagenesis study to explore the functional role of individual domains/motifs in the envelope proteins without potential unwanted cis-effects to virus infection by the introduced mutations in the viral RNA genome. By using this system, we found that the pFP in E1 plays an important role in virus morphogenesis in addition to its well-known contributions to HCV entry (57). The deletion of pFP has no effect on the E1E2 heterodimerization, but completely abolishes the production and release of infectious virions. Alanine scanning analysis identified several point mutations within pFP that specifically affect virus morphogenesis rather than virus fusion (57). These results suggest that the pFP of E1 plays a dual role in virus entry and morphogenesis. More systematical studies are needed to reveal the exact underlying molecular mechanisms and also to investigate the contribution of other domains.

## Conclusion and Perspectives

Hepatitis C virus entry and assembly are complicated process that involves numerous viral proteins and host factors, including E1 and E2. As the conformations of E1 and E2 are interdependent, the functional analysis of each of these two envelope proteins should be always put in the context of the heterodimer. For example, the Ig-fold β-sandwich structure of E2 ectodomain displays similarities with domain III class II fusion proteins (53), suggesting that E2 may serve as a chaperone protein for E1 folding to assist its function in virus fusion. Thus, if we regard the E1/E2 complex as an integrated functional protein, their functions may become easier to understand and characterize.

Compared to E2, E1 is less immunogenic. It is probable that most E1 domains are hidden in the E1E2 heterodimer. However, during the heterodimer conformational changes in virus entry process, some E1 domains must be unmasked to finalize the fusion process. Therefore, the characterization of these dynamically exposed E1 domains, such as structural resolution of the E1E2 complexes in their pre- and post-fusion states, should be the keys to fully understand the roles of E1 in HCV life cycle and to accelerate development of HCV vaccines.

## Author Contributions

YT, JZ, and DL drafted the manuscript; QL performed bioinformatics analysis.

## Conflict of Interest Statement

The authors declare that the research was conducted in the absence of any commercial or financial relationships that could be construed as a potential conflict of interest.

## Abbreviations

HCV, hepatitis C virus; HCVpp, HCV pseudoparticle; HCVcc, cell-culture derived HCV; CLDN1, claudin-1; nE1, N-terminal domain of E1; pFP, putative fusion peptide; C, cysteine; Asn, asparagine; TMD, transmembrane domain.
